# Editorial: Unlearning of Aggressive Behavior and Mechanisms of Change

**DOI:** 10.3389/fpsyt.2022.919122

**Published:** 2022-06-08

**Authors:** Svenja Taubner, Katja Bertsch, Sonja Protić, Thorsten Fehr

**Affiliations:** ^1^Institute for Psychosocial Prevention, University Hospital of the University Heidelberg, Heidelberg, Germany; ^2^Department of Psychology, Ludwig-Maximilians-University Munich, Munich, Germany; ^3^NeuroImaging Core Unit Munich (NICUM), University Hospital LMU, Munich, Germany; ^4^Institute of Criminological and Sociological Research, University of Belgrade, Belgrade, Serbia; ^5^Department of Neuropsychology, Center for Cognitive Sciences, University of Bremen, Bremen, Germany; ^6^Center for Advanced Imaging, Universities of Bremen and Magdeburg, Bremen, Germany

**Keywords:** aggressive behavior, development, treatment, neural correlates, mechanisms

An interesting and astonishing phenomenon in the development of aggression is that there appears to be a lifetime peak of aggressive with behavioral tendencies around two years of age [e.g., (1)]. This observation directly lead to the assumption that aggressive behavior has not to be learned, but rather unlearned during individual socialization [e.g., (2)]. On the other side, aggression must not necessarily be related to violence. Whether an action is of destructive nature depends on contextual and motivational factors, and it is not evident that complex associations between behavioral motives and concrete and complex violent action patterns are inherited (3, 4). It appears most likely that individual schemes of violent behaviors in mentally healthy individuals are a result of complex interactions between environmental, societal, and potentially inherited, temperament-related factors (4–6). However, there is also evidence that neurophysiological disorders and psychopathological diseases can facilitate the development of violent behaviors in manifold ways [e.g., (7–9)].

The present Research Topic outlines a collection of studies that have provided insights into the nature of potential modulators and/or mediators of violent and/or aggressive forms of behavior. These modulators and/or mediators (e.g., pre-, post-, and peri-natal environmental properties, violence-related learning history and socialization, mental illness and psychopathology, as well as communication) can provide puzzle pieces that help us to estimate chances of unlearning destructive and adverse behavioral tendencies.

An et al. reported a relationship between white matter density and aggressive behavioral tendencies in a study with schizophrenic patients. A lack in structural white matter connectivity was related to visuospatial abilities. This finding supports the idea that perceptual handicaps might reduce social signal processing capability in schizophrenic patients. Thus, perceptual training of social signal processing appears to provide an indispensable element in psychiatric treatment of emotionally inadequate behaviors, such as violence. In this sense, Honecker et al. reported success after Mechanism-based Anti-Aggression-Psychotherapy (MAAP) vs. a Non-Specific Supportive Psychotherapy (NSSP) in patients with Borderline Personality Disorder (BPD). MAAP particularly targets mechanisms that were proposed as important factors contributing to reactive aggression in BPD: namely, social threat hypersensitivity, maladaptive anger regulation, approach (rather than avoidance) of social threat cues, low capacity to adequately mentalize others' intentions, cognitions, and emotions, as well as excessive emotional imitation and contagion. Thus, the therapeutic aims included psychoeducation on models of reactive aggression, in addition to the development of inhibition and emotion regulation strategies by using intervention techniques derived from Dialectical Behavior Therapy (DBT) and Mentalization-Based Therapy (MBT). Indeed, perceptual training on finding positive rather than negative social cues produced positive effects in BPD patients. However, subsequent studies on bigger samples that control for additional factors, such as environmental modulators, will ultimately reveal whether these positive effects persist.

Less perceptual, but more complex, mental approaches focused on procedural and executive mental functions and social skill trainings to control impulsive behaviors (10). Talia et al. followed an approach based on emotional empathic skills, such as epistemic trust and its role in the understanding of aggression in conduct disorder. Two propositions were discussed: The first one was to view aggression as a modality of communication adapted to scenarios, in which the communicator expects the audience to have low epistemic trust in the communicator, and the second idea was to conceptualize the failed “unlearning of aggression” as reflecting a lack of interest in maintaining one's reputation as a communicator, which in turn stems from a lack of epistemic trust in other communicators. After integrative discussion, the authors concluded that linking problems in communication to aggression and addressing this as a possible change mechanism may lead to successful further development of therapeutic interventions for the treatment of aggressive youth.

Bertsch et al. disentangled the concepts of an actual frustration-induced anger state from general trait emotion dysregulation. They examined patients BPD and healthy control participants *via* emotional rating scales before and after the performance of the Titrated Mirror Tracing Task (i.e., frustration induction). Before and after the induced frustration, individuals with BPD reported higher levels of negative emotions, however, they further showed a larger increase in anger after frustration-induction (i.e., contextual state modulation). This finding documents the power of context, potentially independent from trait-related factors. In a multi-attribute (i.e., contextual) social decision-making approach, Fehr and Achtziger showed in healthy individuals that reactive behavioral responses as triggered by quasi-realistic scenarios (video stimulation from first person perspective) can be modulated by preceding contextual information (e.g., sentences). Reactive aggressive (i.e., self-defense) responses were stimulated by preceding negative-provocative information in a threatening scenario and reactive pro-social responses were stimulated by preceding social positive context-information. In rather neutral interaction scenarios, negative context information stimulated withdrawal, and positive context information stimulated approach responses. The data point to the importance of contextual properties as modulators of both reactive aggressive (i.e., self-defense) and reactive pro-social behaviors in healthy female and male individuals. Gathering evidence, it appears that in both healthy individuals and individuals with mental disorders (including pathological aggression), contextual features play a substantial role in the modulation of behaviors. Mestre et al. provided an extensive review on potential mediators of externalizing behaviors during adolescence. They concluded that two types of mediators might be involved in the mechanisms of change for better social skill improvements in adolescence: healthier parent–adolescent relationships and parental discipline. Changes in adequate behavioral control of adolescents' peer behavior and a better positive balance in adolescents' relationships with their parents seemed to both explain differences in outcomes in psychotherapeutic intervention for externalizing behaviors in adolescents. The authors encourage the scientific community to put additional efforts into understanding the relationship between internalizing and externalizing symptoms, and furthermore, to consider additional potential factors such as emotional mediators. In general, the conclusions drawn by Mestre et al. fit to the conclusions drawn by Wiechert et al., who compared delinquent individuals with a socialization of pronounced violence with non-delinquent controls without a pronounced violence-socialization in a social decision task using quasi-realistic video stimuli, filmed from a first-person perspective. Participants could choose to approach or to withdraw in threatening, social positive, and neutral situations. In threatening situations, both groups showed periaqueductal (i.e., neural flight, fight, freeze instance) and amygdala (i.e., neural instance for evaluating emotional entities) activations, which points to a healthy basic neural functioning in the processing of threat. As participants with delinquent and violent socialization showed lower middle and inferior frontal (i.e., comprising highly plastic and heteromodal association cortices) activations, the authors concluded realistic perspectives for a successful resocialisation training in delinquent, but basically non-pathological individuals. However, the success of learning-related resocialisation might be diminished by a persistent violent and/or socially incompetent milieu of peers and caregivers (Mestre et al.). As an interesting resocialisation approach, Harwood-Gross et al. discussed the effects of martial arts (MA) training on violence-related cognitive (i.e., inhibition, flexibility, speed of processing, and attention) and psychological (i.e., aggression parameters and self-esteem) functioning in at-risk adolescents. MA training improved both inhibition performance and changed reactive hormonal status (in particular, oxytocin) related to processing speed and aggression, whereas cortisol reactivity was related to increases in self-esteem. In short, the complex contextual intervention by enjoyable group MA training was interpreted as potential mediator of change on factors potentially modulating violent behaviors. Integrating adolescents early in life in sports associations that train both the control of impulses and pro-social skills (i.e., neuronally embedded pro-social perception action concepts) might provide an effective prevention for delinquent careers (cf., Wiechert et al.).

Summarizing, the present Research Topic on the potential mechanisms of “unlearning aggression” provides insights from quite different perspectives, which reveal that there are many internal and external factors contributing to varying degrees of change in aggressive behavioral tendencies. Socialization, to some amount genetically modulated metabolic (i.e., hormonal), neuro-developmental, psychopathological, and many more factors and their interactions shape the individual development of and changes in individual aggressive behavior (4, 11–15). Extensive individual diagnostics and individually adapted interventions appear indispensable for an effective treatment of impulse control distortions. Many more multi-perspective studies need to be realized in order to provide the necessary tools for this mission.

## Author Contributions

TF: writing—original draft. ST, KB, and SP: writing—review and editing. All authors contributed to the article and approved the submitted version.

## Conflict of Interest

The authors declare that the research was conducted in the absence of any commercial or financial relationships that could be construed as a potential conflict of interest.

## Publisher's Note

All claims expressed in this article are solely those of the authors and do not necessarily represent those of their affiliated organizations, or those of the publisher, the editors and the reviewers. Any product that may be evaluated in this article, or claim that may be made by its manufacturer, is not guaranteed or endorsed by the publisher.
